# Hypokalemia-induced Type 1 Brugada Reveals Type 3 Brugada Pattern with Repletion: Case Report

**DOI:** 10.5811/cpcem.47005

**Published:** 2025-07-14

**Authors:** Christina Cantwell, Mark I Langdorf

**Affiliations:** University of California, Irvine, Medical Center, Department of Emergency Medicine, Orange, California

**Keywords:** hypokalemia, Brugada, case report, type 1 Brugada, type 3 Brugada

## Abstract

**Introduction:**

Brugada syndrome is a ventricular arrhythmia and type of sodium channelopathy that can be seen in the absence of structural heart disease. Recognition of this pattern on electrocardiogram (ECG) is important for stabilization and correction of underlying triggers that can be addressed in the emergency department (ED).

**Case report:**

We describe a case of a 58-year-old male who presented with chest pain and was found to have type 1 Brugada pattern in the setting of severe hypokalemia. Repletion of potassium later revealed type 3 Brugada pattern followed by resolution on repeat ECG.

**Conclusion:**

Rapid identification of underlying metabolic derangements that can trigger Brugada syndrome is important in the ED setting. Correction of the underlying abnormality can reveal a type 3 pattern with subsequent resolution of the pattern if well-controlled.

## INTRODUCTION

We describe a case of a 58-year-old male who presented with chest pain and was found to have type 1 Brugada pattern in the setting of severe hypokalemia. Repletion of potassium later revealed type 3 Brugada pattern. Previous case reports have described hypokalemia unmasking type 1 pattern that resolved with electrolyte repletion; here we report type 1 pattern converting to type 3 pattern with normalization of potassium which, to the best of our knowledge, has not yet been reported.

## CASE REPORT

A 58-year-old male of Asian descent with a history of Cushing disease from a pituitary tumor, hypokalemia, and hypertension presented to the emergency department (ED) with one day of chest pain and shortness of breath. His medications included spironolactone, furosemide, metoprolol, potassium supplementation, and amlodipine. There was no known reported family history of cardiac disease.

Upon arrival the patient was initially an ST-elevation myocardial infarction (STEMI) activation from triage. Initial vitals were temperature 37.1 °C, heart rate 93 beats per minute, blood pressure 158/94 millimeters of mercury, and oxygen saturation 100% on room air with respiratory rate of 18 breaths per minute. He was alert and in no acute distress. Cardiac exam was significant for regular rate and rhythm with equal bilateral peripheral pulses and no audible murmur. Lungs were clear to auscultation bilaterally. There was symmetric 3+ pitting edema to the knees bilaterally.

**Image 1:**
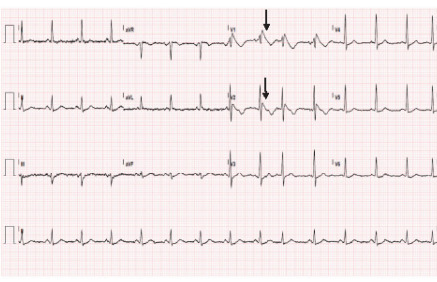
Initial electrocardiogram on arrival showing type 1 Brugada pattern in a patient presenting with chest pain. Note the straightening of the ST segment from the QRS complex to the inverted T wave (arrows).

Differential diagnosis included acute coronary syndrome, arrhythmia, Brugada syndrome, electrolyte derangement, aortic dissection, pulmonary embolism, and pneumonia. Workup initially included complete blood count, comprehensive metabolic panel, thyroid panel, serum troponin I, D-dimer, magnesium level, B-type natriuretic peptide, prothrombin time/international normalized ratio/partial thromboplastin time, hemoglobin A1c, venous blood gas, repeat electrocardiogram, portable one-view chest radiograph, and cardiology consult. Labs were significant for hypokalemia of 2.5 millimoles per liter (mmol/L) (reference range: 3.5–5.1 mmol/L), troponin of 40 nanograms (ng)/L (0–20 ng/L), glucose of 356 milligrams per deciliter (mg/dL) (70–115 mg/dL). Electrocardiogram (ECG) showed type 1 Brugada pattern (Image 1) with an incomplete right bundle branch block pattern in V1 and V2 followed by straightening of the ST segment from the top of the QRS complex leading to an inverted T wave. Chest radiograph revealed a widened mediastinum, and computed tomography angiogram of the whole aorta ruled out aortic dissection. Diagnostic challenges included hypokalemia precluding the patient from being optimized for potential cardiac catheterization. However, when the type 1 Brugada pattern was recognized in the ED, the STEMI activation was cancelled.

Potassium repletion initiated in the ED consisted of 40 milliequivalents (mEq) oral and 40 mEq intravenous potassium chloride. Chest pain improved with two sublingual sprays of nitroglycerin, 400 micrograms each, and with potassium repletion.

**Image 2:**
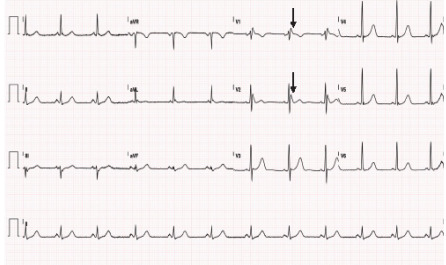
Second electrocardiogram 24 hours after arrival showing type 3 Brugada pattern after correcting severe hypokalemia. Note the ST segment in leads V1 and V2 are now less than two millimeters (arrows).

After cardiology consultation, the patient was admitted to the internal medicine service for further workup and cardiac monitoring. Troponin downtrended to 33 ng/L on repeat check one hour later. Upon repletion of potassium, repeat ECG 24 and 48 hours after initial presentation revealed type 3 Brugada patterns (Image 2).

**Image 3:**
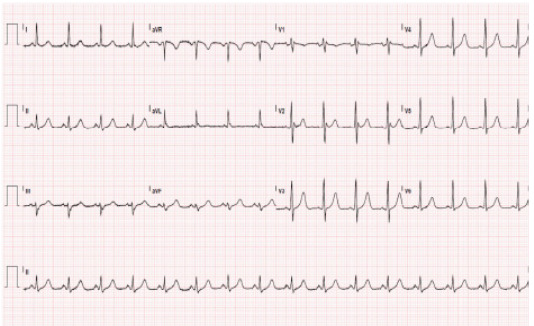
Electrocardiogram 48 hours later showing normal sinus rhythm and no indication of Brugada pattern after achieving normalization of electrolytes.

During his hospital course, the patient had difficult-to-control hypokalemia and hyperglycemia concerning for a hypercortisol state with an unclear source despite unremarkable magnetic resonance imaging and positron emission tomography without identified tumor or mass, ultimately requiring initiation of insulin and aggressive potassium supplementation with potassium chloride 40 mEq three times daily. Forty-eight hours and six weeks after initial presentation, his ECG showed normal sinus rhythm without Brugada pattern (Image 3). (Six-week ECG is not shown).

*CPC-EM Capsule*

What do we already know about this clinical entity?

*Brugada syndrome is a sodium channelopathy that can predispose to life-threatening arrhythmias if left untreated*.

What makes this presentation of disease reportable?

*In a patient with severe hypokalemia, Brugada pattern is seen to transform from type 1 to type 3 pattern and ultimately resolve with electrolyte repletion*.

What is the major learning point?

*A hypokalemic state can cause a hyperpolarization of the cardiac membrane and slow the action potential resulting in a reversible Brugada pattern when corrected*.

How might this improve emergency medicine practice?

*Identification and correction of underlying potassium abnormality can reveal a type 3 pattern with subsequent resolution of the Brugada pattern if well-controlled*.

## DISCUSSION

Brugada syndrome has been reported as a type of ventricular arrhythmia identified in patients in the absence of known structural heart disease.^1^ This pattern was initially characterized by a right bundle branch block and ST-segment elevation in V1, V2, and V3 leads, which our patient had on arrival. The first study by Brugada and Brugada in 1992 found this pattern in the absence of electrolyte derangement.^1^ It is now known that Brugada syndrome is a sodium channelopathy caused by a genetic mutation in the sodium channel gene.^2^ Factors that can unmask a Brugada pattern include fever, certain drugs, hypokalemia, hyperkalemia, and hypothermia.^2^ Type 1 describes an incomplete right bundle branch block pattern in V1 and V2 followed by straightening of the ST segment from the top of the QRS complex leading to an inverted T wave. Type 2 describes a saddleback ST-segment pattern, and type 3 describes type 1 or type 2 morphology but with < 2 mm of ST-segment elevation. While there have been case reports of a type 1 pattern being seen with hypokalemia that resolves with repletion,^3^,^4^,^5^ in this case a type 3 pattern was revealed as potassium normalized.

In the cardiac myocyte, a hypokalemic state can cause hyperpolarization of the membrane and inhibit the Na^+^-K^+^ ATPase, resulting overall in increased intracellular sodium and calcium and a slowed action potential.^6^ While types 2 and 3 patterns are suggestive of Brugada disease, the type 1 pattern is diagnostic.^7^ In this case, normalization of serum potassium levels reduced and ultimately resolved the ST-segment Brugada patterns. The type 3 pattern may be more favorable physiologically compared to type 1 given that the ST-segment elevation is less pronounced, representing a less delayed action potential.

## CONCLUSION

Brugada pattern can be seen in severe underlying metabolic derangement—in this case, associated with hypokalemia. It is important to identify and address any triggers such as metabolic derangement and infection and to avoid drugs that affect serum potassium levels. Correction of the underlying abnormality can reveal a type 3 pattern with subsequent resolution of the pattern if well-controlled. While our patient did not have any resultant arrhythmias characteristic of Brugada syndrome, he did have the electrocardiographic changes consistent with Brugada pattern.
